# Effects of *Hitoegusa* (*Monostroma nitidum*) Extracts on In Vitro Rumen Fermentation Profiles

**DOI:** 10.1111/asj.70117

**Published:** 2025-10-06

**Authors:** Naoya Okada, Tomomi Ban‐Tokuda, Hiroki Matsui

**Affiliations:** ^1^ Graduate School of Bioresources Mie University Tsu Mie Japan

**Keywords:** ruminants, *Hitoegusa* (*Monostroma nitidum*) extract, in vitro rumen fermentation, rhamnan sulfate

## Abstract

In vitro rumen fermentations were conducted using extracts of the seaweed *Hitoegusa* (*Monostroma nitidum*), which contains rhamnan sulfate, as a fermentation substrate. This study examines the metabolic utilization of *Hitoegusa* extract by rumen microbiota through in vitro fermentation assays. In the first incubation, the addition of *Hitoegusa* extract led to increased gas production and total short‐chain fatty acid (SCFA) concentrations, indicative of enhanced microbial activity and substrate fermentation. Alterations in SCFA profiles suggest a potential modulation of the rumen microbial community. In the second incubation, the incubation period was extended to 96 h, and gas production was measured. In any incubation period, gas production was increased in a dose‐dependent manner. Supplementation with *Hitoegusa* extract to wheat starch did not result in a significant reduction in methanogenesis, possibly due to the limited biodegradability of rhamnan sulfate or an insufficient release of sulfate moieties. Further investigations are required to evaluate the degradability and bioavailability of rhamnan sulfate, isolate rumen bacteria capable of degrading this polysaccharide, and characterize the relevant enzymatic and genetic mechanisms. This study highlights the potential of underutilized marine macroalgae, such as *Hitoegusa*, as alternative feed resources for ruminant nutrition.

## 1 Introduction

The edible seaweed *Hitoegusa* (*Monostroma nitidum*) is a green alga classified within the family Monostromaceae. It contains high concentrations of the sulfated polysaccharide rhamnan sulfate (Nakamura et al. 2011), which functions as an intercellular structural component. Rhamnan sulfate is composed of a principal backbone of five α‐1,3‐linked L‐rhamnose residues, with two α‐1,2‐linked L‐rhamnose residues attached at the C‐2 position as side chains, along with a β‐D‐glucuronic acid moiety at the nonreducing terminus. Additionally, sulfate groups are incorporated as further structural modifications.

Unlike terrestrial plants, which possess cell walls rich in recalcitrant lignin, seaweeds and seagrasses lack lignin in their cell walls, thereby facilitating the efficient degradation of structural polysaccharides.

The North Ronaldsay breed of sheep, native to the Orkney Islands, Scotland, primarily consumes seaweed that washes ashore (Orpin et al. 1985). These sheep endure extreme coastal conditions, relying almost exclusively on seaweed as their primary dietary resource (Hansen et al. 2003). The ruminal microbiota of these sheep has adapted to the degradation of seagrass polysaccharides. *In sacco* and in vitro assessments concluded that *Ulva lactuca* represents a medium‐quality forage for goats (Ventura and Castanon 1998). Williams et al. (2012) successfully isolated bacterial strains from their rumen capable of metabolizing complex polysaccharides such as carrageenan, fucoidan, and laminarin, which are integral to seagrass cell walls.

Rhamnan sulfate exhibits a diverse array of bioactive properties, including anticoagulant, thrombolytic, antiviral, antiobesity, and anti‐inflammatory activities, rendering it a promising candidate for applications in the food and pharmaceutical industries (Chi et al. 2023; Terasawa et al. 2022). However, in contrast to other seaweed‐derived polysaccharides such as alginate, carrageenan, and fucoidan, rhamnan sulfate has received relatively limited research attention.

In ruminants, ingested feed first undergoes microbial degradation and fermentation within the rumen. Clinical trials have demonstrated that rhamnan sulfate supplementation modulates gut microbiota composition, leading to a reduction in Firmicutes and Clostridia abundance, while promoting an increase in Acidaminococcales and Veillonellales populations (Shimada et al. 2021). More recently, rhamnan sulfate was shown to significantly alter the gut microbiota composition in mice, increasing the relative abundances of Prevotellaceae UCG‐001, Clostridia vadinBB60 group, and *Mucispirillum schaedleri* , while decreasing Rikenellaceae RC9 gut group, ASF356, and *Staphylococcus* (Tochitani et al. 2025). These findings suggest the potential of rhamnan sulfate to modulate ruminal microbiota in ruminants; however, no studies have yet investigated its effects on rumen fermentation.

In vitro rumen fermentation trials have demonstrated that the inclusion of seaweeds such as *Ulva* sp., *Gigartina* sp., and *Gracilaria vermiculophylla* with meadow hay significantly reduces data production (Maia et al. 2016). The macroalgae *Asparagopsis taxiformis* and *Asparagopsis armata* , when incorporated at low inclusion rates into the diets of cattle and sheep, suppress methanogenesis by up to 98%, accompanied by evidence indicating enhanced feed conversion efficiency (Glasson et al. 2022). A principal bioactive compound responsible for this methane mitigation is bromoform. Li et al. (2025) conducted metagenomic analyses using in vitro rumen fermentation systems to elucidate the underlying mechanisms. Supplementation with low levels of freeze‐dried *A. taxiformis* significantly elevated the relative abundance of propionate‐producing microbial taxa and augmented propionate biosynthesis. This caused reducing equivalents to be incorporated into propionate, thereby decreasing hydrogen availability for methanogenesis. Moreover, Kyoto Encyclopedia of Genes and Genomes (KEGG) pathway analysis revealed that *A. taxiformis* markedly suppressed the abundance of the methyl‐coenzyme M reductase alpha subunit, thereby directly inhibiting methane (CH₄) biosynthesis. These results suggest that seaweed‐derived compounds could serve as potent ruminal methane mitigation agents.

In this preliminary study, we aimed to evaluate the effects of *Hitoegusa* extract containing rhamnan sulfate on in vitro rumen fermentation dynamics.

## 2 Materials and Methods

### 2.1 Ruminal Contents Donor

The animal care and sampling procedures were approved by the Institutional Animal Care and Use Committee of Mie University (Approval Number: 2024‐38) and were performed according to the Mie University guidelines for laboratory animals. Three ruminally cannulated female crossbred cattle (Holstein × Japanese Black cattle; mean body weight: 608 kg) were used as donors of ruminal contents. The animals were fed 4 kg of commercial concentrate and 4 kg of Italian ryegrass per day, divided into two equal portions and administered at 10:00 and 17:00. Water and mineral supplements were provided ad libitum.

### 2.2 In Vitro Culture

A 50‐mL vial was used as the incubation vessel. *Hitoegusa* extract (comprising 70% rhamnan sulfate, 20% cellulose, and 10% protein), either alone or in combination with wheat starch, served as the fermentation substrate. To evaluate the dosage effect of the extract, 0 g (Control), 0.025 g, 0.050 g, 0.125 g, or 0.250 g of the extract was supplemented as the sole substrate. For methane production analysis, all treatments included 0.125 g of wheat starch, with the addition of 0 g (Control), 0.025 g, 0.050 g, 0.125 g, or 0.250 g of the extract.

Rumen fluid was collected from the fistulated cattle prior to the morning feeding and transported to the laboratory while maintained at 39°C. The fluid was filtered through four layers of surgical gauze and diluted threefold with McDougall's buffer (McDougall 1948), which had been prewarmed to 39°C and continuously flushed with nitrogen gas. The vials were subsequently inoculated with 12.5 mL of the diluted rumen fluid (*n* = 3 per animal). Following inoculation, the vials were tightly sealed with butyl rubber stoppers and aluminum crimp seals and then incubated at 39°C. All procedures, from rumen fluid dilution to dispensing, were conducted under strict anaerobic conditions by maintaining a continuous nitrogen gas flow. The first incubation for only *Hitoegusa* extract had an incubation period of 24 h. In the second incubation for only *Hitoegusa* extract, the incubation period was extended to 24, 48, 72, and 96 h. The third incubation period was 24 h for wheat starch and *Hitoegusa* extract. Because the measurement of methane production is performed over a relatively short incubation period (Sarwono et al. 2019), a 24‐h incubation period was adopted. To obtain methane production from the third incubation, gas production and gas concentration were determined as described in the next section.

### 2.3 Determination of Gas Production and Gas Concentration

In the third incubation, gas production in the headspace of the vials during in vitro incubation was quantified using a gas pressure gauge (Aφ60B, GL Science, Tokyo, Japan) (Watanabe et al. 2010). Following pressure measurement, the headspace gas composition was analyzed via gas chromatography (GC‐8AIT, Shimadzu, Kyoto, Japan), equipped with a Shincarbon ST column (3‐mm inner diameter, 2‐m length) and a thermal conductivity detector, as described by Manlapig et al. (2024). The column temperature was maintained at 100°C, while the detector temperature was set at 210°C. High‐purity argon was used as the carrier gas at a flow rate of 50 mL/min. A 0.5‐mL aliquot of headspace gas was injected into the gas chromatograph using a gas‐tight syringe. Peak identification and gas concentration (%) determination were conducted using an integrator (C‐R8A, Shimadzu, Kyoto, Japan).

### 2.4 Measurement of Short‐Chain Fatty Acids

Following the quantification of gas production and composition, a 1‐mL aliquot of culture fluid was collected for short‐chain fatty acid (SCFA) analysis and preserved at −30°C. SCFA concentrations in the culture fluid were determined using high‐performance liquid chromatography (HPLC), as described by Matsui et al. (2013).

### 2.5 Statistical Analysis

Statistical analyses were conducted using IBM SPSS Statistics Version 26. For gas production data, a two‐way analysis of variance (ANOVA) followed by Dunnett's test was applied. For SCFA production and composition, as well as methane production data, a one‐way ANOVA followed by Tukey's test was employed. A probability value of < 0.05 was considered statistically significant.

## 3 Results and Discussion

Seaweed is recognized as a promising and valuable feed resource, with its commercialization anticipated in the near future (Halmemies‐Beauchet‐Filleau et al. 2018). Specifically, it has been identified as a suitable feed for ruminants (Belanche et al. 2016). While various seaweed species have been investigated as feed sources for ruminant livestock (Makkar et al. 2016), no studies have been conducted on *Hitoegusa*. This study constitutes the first report on the utilization of *Hitoegusa* extract by rumen microorganisms.

**FIGURE 1:**
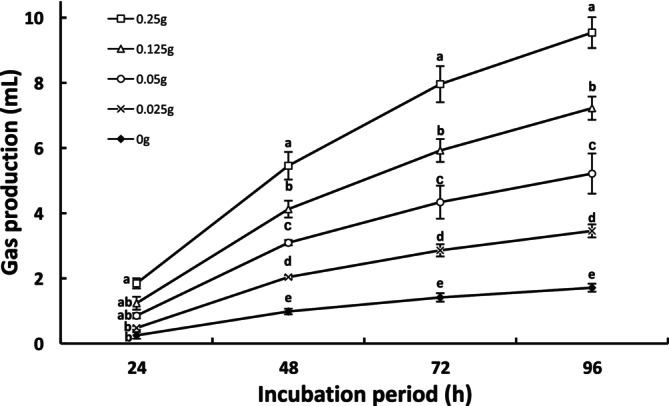
The effect of dosage of *Hitoegusa* extract on gas production in in vitro rumen fermentation after 24‐, 48‐, 72‐, and 96‐h incubation.. Values are expressed as mean ± SE (*n* = 3).. ^a, b, c, d, e^Mean values with different superscripts at same incubation period are significantly different (*p* < 0.05).

An in vitro rumen culture was performed using *Hitoegusa* extract as the sole substrate to evaluate the extent of its utilization and fermentation. The first incubation with only *Hitoegusa* extract, gas production, and total SCFA concentration and composition were measured after 24 h of incubation (Table 1). The gas production was higher with *Hitoegusa* extract addition than in the control. Furthermore, the gas production was increased in a dose‐dependent manner. Total SCFA concentration was significantly higher at the 0.25 g dosage of *Hitoegusa* extract. Moreover, the proportion of acetic acid decreased significantly with increasing levels of *Hitoegusa* extract, whereas the proportion of propionic acid increased significantly (Table 1). These findings confirm active fermentation in this culture system and suggest the presence of microbial populations capable of utilizing and metabolizing the components of *Hitoegusa* extract. To clarify this, isolation of related microorganisms is required. In addition, degradation and utilization of *Hitoegusa* extract by the isolated microorganisms should be examined. In the second incubation, gas production at 24, 48, 72, and 96 h after incubation was measured as an indicator of microbial fermentation and substrate utilization (Figure 1). At all incubation periods, gas production was significantly higher than in the control group (0‐mg dosage), where no substrate was provided, confirming the fermentation of *Hitoegusa* extract components by rumen microorganisms. Furthermore, gas production exhibited a dose‐dependent increase.

**TABLE 1:** The effect of *Hitoegusa* extract on gas production and short chain fatty acid in in vitro rumen fermentation after 24‐h incubation

Fermentation parameters	*Hitoegusa* dosage (g)				
	0.000	0.005	0.025	0.125	0.250
Gas production (mL)	0.31^e^ ± 0.01	0.65^d^ ± 0.03	0.80^bc^ ± 0.08	0.84^b^ ± 0.09	1.03^a^ ± 0.01
Total short‐chain fatty acids (mmol/L)	34.5^b^ ± 3.8	36.1^b^ ± 3.4	39.8^ab^ ± 3.9	38.9^ab^ ± 3.2	44.8^a^ ± 2.5
Acetic acid (%)	76.2^a^ ± 1.0	72.2^b^ ± 0.9	72.5^b^ ± 0.8	71.1^b^ ± 0.8	68.0^c^ ± 0.4
Propionic acid (%)	11.8 ^a^ ± 0.9	13.1^b^ ± 0.9	13.8^b^ ± 0.6	16.2^c^ ± 0.7	20.0^d^ ± 1.0
Butyric acid (%)	12.1^a^ ± 1.1	14.7^a^ ± 2.2	13.7^a^ ± 0.3	12.7^a^ ± 0.7	12.1^a^ ± 1.2

*Note:* Values are expressed as mean ± SE. Mean values with different superscripts (^a, b, c, d, e^) within a row are significantly different (*p* < 0.05) (*n* = 3)

Orpin et al. (1985) examined the microflora of seaweed‐fed sheep and reported a predominance of the ciliate protozoan *Dasytricha ruminantium*. In contrast, no rumen fungi or cellulolytic bacteria were detected, with dominant bacterial species including *Streptococcus bovis* , *Selenomonas ruminantium* , and *Butyrivibrio fibrisolvens* . Although microbial composition was not analyzed in the present study, the observed alterations in SCFA composition following *Hitoegusa* extract supplementation suggest a potential shift in the rumen microflora. Since the propionate composition is increased in the supplementation of *Hitoegusa* extract, abundance of propionate‐producing bacteria could be expected.

**FIGURE 2:**
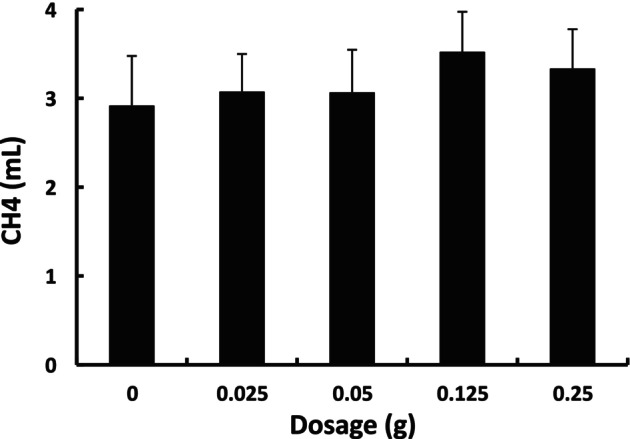
The effect of dosage of *Hitoegusa* extract on methane production (mL) in in vitro rumen fermentation after 24‐h incubation. Values are expressed as mean ± SE (n = 3).

The impact of *Hitoegusa* extract on methanogenesis in in vitro rumen culture was also evaluated in the third incubation. Rhamnan sulfate, a major constituent of *Hitoegusa* extract, contains sulfate groups. If sulfate ions from rhamnan sulfate are released into the culture medium, they may serve as electron acceptors for sulfate‐reducing bacteria, thereby potentially suppressing methanogenesis (Zhao and Zhao 2022). To investigate this possibility, methanogenesis was assessed following 24 h of incubation, using wheat starch as the primary substrate with the addition of *Hitoegusa* extract (Figure 2). However, no significant reduction in methane production was observed with any level of *Hitoegusa* supplementation. This lack of effect may be attributed to the limited degradation of rhamnan sulfate or the insufficient release of sulfate groups, preventing hydrogen utilization via sulfate reduction (van Zijderveld et al. 2010). The enzymatic capacity of rumen microorganisms to release sulfate groups from rhamnan sulfate remains unknown, as no studies have reported such activity. Therefore, future research should focus on assessing the enzymatic activity involved in sulfate group release by rumen microorganisms. Previous studies have demonstrated methane mitigation by various seaweed species in in vitro rumen cultures (Maia et al. 2016; Molina‐Alcaide et al. 2017). However, the extent of methane suppression has varied across seaweed species, exhibiting no consistent trend.

Given that impure substrates were used in the present study, future research should focus on utilizing purified rhamnan sulfate as a substrate to determine its degradability and bioavailability, thereby clarifying the extent to which rumen microorganisms can metabolize rhamnan. Additionally, efforts should be directed toward isolating rhamnan sulfate‐degrading bacteria from the rumen. If such isolates are identified, biochemical characterization of the rhamnan sulfate‐degrading enzymes they produce, along with the cloning of the corresponding genes, will be essential. Furthermore, the potential of rhamnan‐containing seaweeds as ruminant feed resources warrants further evaluation.

## Conflicts of Interest

The authors declare no conflicts of interest.
